# Clustering of Obesity-Related Risk Behaviors Among Families With Preschool Children Using a Socioecological Approach: Cross-Sectional Study

**DOI:** 10.2196/10320

**Published:** 2018-04-25

**Authors:** Virginia Quick

**Affiliations:** ^1^ Department of Nutritional Sciences Rutgers University New Brunswick, NJ United States

**Keywords:** obesity, family, preschool children, socioecological, risk factors, environment, home, physical activity, screen time

## Abstract

**Background:**

Limited attention has been given to assessing home environments of parents with preschool-aged children using a socioecological approach to better understand potential influencers of obesity risk.

**Objective:**

The purpose of this cross-sectional study was to examine the clustering of obesity-related risk behaviors among mothers with preschool children.

**Methods:**

Mothers with preschool-aged children (ages 2 to 5 years) who participated in the online Home Obesogenic Measure of Environments (HOMES) survey were examined in clustering of four healthy recommended behaviors (ie, mother’s fruit and vegetable intake ≥5 per day, sedentary screen time <4 hours per day, sugar-sweetened beverage intake <1 time/day, and increased physical activity level). Frequencies and percents of the clustering variables were conducted along with Spearman rank order correlations to determine significant associations. Ward’s method with squared Euclidean distances were performed for the cluster analysis using the four standardized continuous variables. Identification of total cluster number was determined by visually inspecting the dendogram. Sociodemographic, intrapersonal, social environment, and home physical environment characteristic differences between cluster groups were further examined by independent t tests and chi-square analysis to validate findings.

**Results:**

Of the 496 participants (72.6%, 360/496 white; age mean 32.36, SD 5.68 years), only a third (37.1%, 184/496) consumed five or more servings of fruits/vegetables daily, had low sedentary screen time of <4 hours/day, and reported moderate to high levels of physical activity (34.1%, 169/496). More than half (57.7%, 286/496) consumed <1 sugar-sweetened beverage serving daily. A positive correlation (r=.34, *P*<.001) between physical activity level and fruit/vegetable intake (≥5 servings/day), and a positive correlation (r=.15, *P*=.001) between low sedentary screen time (<4 hours/day) and low sugar-sweetened beverage intake (<1 serving/day) were found. Ward’s hierarchical analysis revealed a two-cluster solution: less healthy/inactive moms (n=280) and health conscious/active moms (n=216). Health conscious/active moms were significantly (*P*<.010) likely to be more physically active, have lower sedentary screen time, lower daily intake of sugar-sweetened beverages, and greater daily intake of fruits and vegetables compared to less healthy/inactive moms. Less healthy/inactive moms were significantly more likely to have a higher body mass index and waist circumference compared to the other cluster; however, there were no significant sociodemographic differences. There were many intrapersonal (eg, importance of physical activity for child and self) and home physical environment (eg, home availability of fruits/vegetables and salty/fatty snacks) characteristic differences between clusters, but few significant differences emerged for social environment characteristics (eg, family meals, family cohesion).

**Conclusions:**

Findings may have implications in tailoring future obesity prevention interventions among families with young children.

## Introduction

In the United States, more than one-third of children are either overweight or obese [1]. The high prevalence rate among all pediatric age groups, in both sexes, and in various ethnic and racial groups has been at a steady climb over the last three decades and this trend continues to this day [2]. Additionally, the overall estimated annual medical costs and physical and mental health consequences of obesity are very high [3].

An energy imbalance in which too few calories are expended for the amount of calories consumed is often the primary focus of obesity research and interventions; however, the many intrapersonal and environmental (social and physical) factors facilitating this energy imbalance are critical to understand. The socioecological model posits that health and well-being of an individual is determined by multiple levels of influence [4]. At the macro level, factors such as economic policies and political and legal structures (eg, gross domestic product) have a more indirect effect in influencing behaviors. At the micro level, factors of the near physical environment (eg, home, neighborhood), family social environment, and intrapersonal characteristics more directly influence behaviors.

Application of ecological theory in obesity research has indicated that intrapersonal characteristics, social environments, and physical environment factors all play a role in obesity [5]. Environments that lack support for weight-management behaviors make it difficult for individuals to engage in behaviors that prevent weight gain. Currently, obesity prevention interventions in children younger than 5 years of age have shown limited effectiveness in reducing or limiting weight gain [6], perhaps due to little attention being given to the social and physical environments within which diet and physical activity behaviors are endorsed [7].

The research to date on the prevention and treatment of obesity among children and adults highlights the importance of increased consideration of the social and physical environment [8]. At the micro level, the home environment is shared among parents and their children. Parents are considered the “gate keepers” of the home and role models for their children. That is, parents can strongly influence food and physical activity behaviors and practices which, in turn, may influence their child’s obesity risk [9,10]. Prior research has reported a number of parent and social and physical environment factors in the home associated with children’s overweight status, such as limited physical activity supports [11], infrequent family meals [12], low household availability of fruits and vegetables [13], excessive sedentary screen time [12], and less parental modeling of healthy behaviors [14].

Mothers, in particular, can have a strong influence on their child’s weight-related behaviors from a young age that develop during the preschool years and track later into childhood and adulthood [15-19]. Given the home environment may influence child obesity risk, it is important to better understand these factors. Limited attention has been given to comprehensively assess the home environment of parents with preschool-aged children using a socioecological approach with reliable and validated measures, which is necessary for better understanding the potential influencers of obesity risk among families with young children [20]. Thus, a secondary analysis from a rich dataset of socioecological factors related to the obesogenic home environments of mothers with preschool-aged children (2 to 5 years of age) was examined to assess the clustering of obesity-related behaviors.

## Methods

The Institutional Review Board at Rutgers University approved this research study. All participants gave informed consent to participate.

### Recruitment

A global research company (ie, Survey Sampling International) whose services include survey participant recruitment, sent invitations to panel members who were mothers in the United States, inviting them to complete the online Home Obesogenic Measure of Environments (HOMES) survey [4,21,22]. Recruitment notices asked mothers to participate in a survey to help researchers “learn more about families with young kids” and to help them develop “a program for parents to build healthier kids.” To be eligible, panel members had to be female, 18 to 45 years of age, English speaking, have at least one preschool child (aged 2 to 5 years), and be the main household food gatekeeper (ie, make most or all food purchasing and meal decisions). Participants and their spouse/partner could not be employed in a health-related profession. As an incentive to complete the survey, participating mothers accrued points from Survey Sampling International that were redeemable for gifts.

### Instruments

Details on the development and content of the online HOMES survey and research protocol are described elsewhere [4,21,22]. In brief, the HOMES cross-sectional survey was developed by researchers at Rutgers University as part of a larger research study exploring obesity risk in mothers of young children and included an array of valid, reliable measures that focused on mother’s sociodemographic, health-related, intrapersonal, social, and home physical environment characteristics. Measures were selected to yield an understanding of socioecological factors pertaining to diet and physical activity. All measures were self-reported and underwent rigorous selection to ensure they were valid and reliable. The survey was posted online using Qualtrics platform and pilot-tested with 48 participants to gauge completion time, identify further refinements needed to improve clarity and ease of completion, ensure protocols for scoring of scales were accurate, and conduct preliminary psychometric analyses. Administration of an online format was chosen for ease of data collection and convenience to participants, to help reduce the potential for social desirability bias that can occur during in-person administration, and to increase researcher ability to reach individuals who would be otherwise difficult to access (ie, distance from researchers or limited time to meet in-person). Multimedia Appendix 1 lists the variables used in this secondary analysis of the online survey including number of items, possible score range, scale type, and internal consistency (Cronbach alpha when applicable).

#### Sociodemographics and Health-Related Characteristics

Maternal sociodemographic data collected included race/ethnicity, highest education level achieved, number of children in the household, family affluence [23,24], and food insecurity risk [25]. Health-related characteristics assessed were general health status (Centers for Disease Control and Prevention [CDC] Health-Related Quality of Life) [26,27], depression severity (Patient Health Questionnaire-2) [28], body dissatisfaction (Eating Disorder Examination Questionnaire) [29], and primary relative with history of obesity.

#### Weight Status and Waist Circumference

Mothers reported their current height, weight, and waist circumference. Height and weight were used to calculate body mass index (BMI) as recommended by the CDC [30]. Mothers reported their child’s height and weight, which were used to calculate age- and sex-specific child BMI percentile.

#### Intrapersonal Characteristics

Mothers’ weight-related behaviors assessed were physical activity level (streamlined International Physical Activity Questionnaire) [31-33] and sleep quality and duration (Pittsburgh Sleep Quality Index) [34,35]. Maternal dietary intake was assessed using the following food frequency questionnaires: Block Fruit-Vegetable-Fiber Screener, Block Dietary Fat Screener [36-38], and a sugar-sweetened beverage screener [39]. Maternal eating styles measured from the Three-Factor Eating Questionnaire-18 [40,41] were disinhibited eating, emotional eating, and dietary restraint eating. Mothers’ self-perceptions assessed were personal organization [42], need for cognition [43,44], parenting self-efficacy [45,46], stress management [47], and stress management self-efficacy (created de novo). Value of engaging in healthy behaviors for self and child (eg, encouragement and facilitation of children’s physical activity, importance of modeling physical activity to children, frequency of engaging in active play with children, parent modeling healthy eating) [11,21,48-50] were also measured.

#### Social Environment

Family meal patterns and family meal environment (eg, frequency of meals, family meal atmosphere) [11,51-55] data were collected. Scales assessing family functioning and engagement included family conflict and lack of cohesion [56-58], and family support for healthy behaviors [59-61].

#### Home Physical Environment

Evaluation of the home environment’s accessibility and availability to physical activity and sedentary activity supports (eg, media devices in the home, TV accessibility for child) [11,48-50,60,62,63] were assessed along with measures of household food availability (eg, fruit/vegetables, sugar-sweetened beverages) [36,39,64-66].

### Obesity-Related Behaviors

It was decided *a priori* that healthy recommended behaviors, such as mother’s fruit and vegetable intake of five or more per day, sedentary screen time (<4 hours per day), sugar-sweetened beverage intake (<1 time/day), and increased physical activity level, would be the variables used in clustering mother’s obesity-related behaviors. Currently, the Dietary Guidelines for Americans [67] recommend adults consume 5 to 9 servings of fruits and vegetables per day and limit the amount of daily sugar-sweetened beverage consumption; thus, a cut-off of five or more fruits and vegetables per day and less than one serving of sugar-sweetened beverage intake per day, respectively, were proxies for healthy behaviors. Although the American Academy of Pediatrics recommends parents monitor and limit their preschool child’s screen time to less than 1 hour per day, there are no set time limits for adults [68]. For this reason, a liberal approach was taken in giving a cut-off of less than 4 hours daily of sedentary screen time for mothers. Physical activity levels, using the streamlined International Physical Activity Questionnaire [31-33], were categorized into low, moderate, and high levels using cut-off scores previously set by other researchers [31,33]. That is, self-report physical activity level was calculated as: (number of days walking per week) + 2 * (number of days moderate-intensity activities per week) + 3 * (number of days vigorous-intensity activities per week), with a possible score range of 0 to 42 (categorized as low: 0 to <20; moderate: 20 to <30; high: ≥30 physical activity levels).

### Statistical Analysis

Frequencies and percents of clustering variables were examined to describe the sample of mother’s meeting these defined fruit and vegetable intake, sugar-sweetened beverage intake, sedentary screen time, and physical activity level behaviors. Before clustering, correlations among the four cluster variables were examined to determine significant associations using Spearman rank order correlations. For cluster analysis, the clustering variables were standardized (*z* scores) to permit comparisons of means and variances [69]. The four clustering variables (ie, mother’s physical activity level, sedentary screen time, daily fruit and vegetable intake, and sugar-sweetened beverage intake) were considered as continuous variables in the model. Ward’s method with squared Euclidean distances was used for the cluster analysis using the standardized continuous variables mentioned previously. Ward’s method was used because it has yielded useful results in previously similar settings [70], and tends to result in clusters of more equal size, which increases the robustness of cross-cluster comparisons [71]. The number of clusters that emerged was identified by visually inspecting the dendogram and noting the point at which the scree graph angled most sharply upward.

To further establish how the two clusters differed from one another, variables used in the cluster analysis as well as intrapersonal, social environment, and home physical environment variables not used in defining the clusters, were examined using independent *t* tests for continuous variables and chi-square analysis for categorical variables. Given the large number of tests, *P* values were reduced to *P*<.010 to be considered statistically significant. All analyses were performed using SPSS version 24.

## Results

### Participant Characteristics

A total of 496 participants (72.6%, 360/496 white; age mean 32.36, 5.68 SD years) with complete and plausible data were included in the analyses (48 had implausible numbers for sedentary screen time [≥15 hours per day] and three each had missing data for daily fruit/vegetable servings and sugar-sweetened beverage intake). More than one-third of participants (37.1%, 184/496) met daily fruit and vegetable serving recommendations of five or more and had relatively low sedentary screen time of less than 4 hours per day (Table 1). More than half of participants (57.7%, 286/496) consumed less than one sugar-sweetened beverage serving daily. Sugar-sweetened beverages included soft drinks, fruit drinks, energy drinks, and sugar-sweetened specialty coffee drinks. Additionally, approximately one-third (34.1%, 169/496) of mothers reported moderate to high levels of physical activity in the last week.

Spearman rank order correlations revealed a positive correlation (*r=*.34, *P*<.001) between physical activity level and fruit and vegetable intake (≥5 servings/day), and a positive correlation (*r=*.15, *P=*.001) between low sedentary screen time (<4 hours/day) and low sugar-sweetened beverage intake (<1 serving/day; Table 2).

### Cluster Group Characteristics

Ward’s hierarchical analysis revealed a two-cluster solution for the participants using the four standardized measures (ie, mother’s physical activity level, sedentary screen time, daily fruit and vegetable intake, and sugar-sweetened beverage intake). The two clusters of mothers were broadly divided as less healthy/inactive moms (n=280) and health conscious/active moms (n=216).

**Table 1:** Proportions of mother’s fruit and vegetable intake, sedentary screen time, sugar-sweetened beverage intake, and physical activity level (N=496).

Description		n (%)
**Fruit/Vegetable intake (≥5 servings/day)**		184 (37.1)
	0 to <2 servings	51 (10.3)
	2 to <3 servings	72 (14.5)
	3 to <4 servings	72 (14.5)
	4 to <5 servings	117 (23.6)
	5 to 6 servings	79 (15.9)
	≥6 servings	105 (21.2)
**Sedentary screen time (<4 hours/day)**		186 (37.5)
	0 to 2 hours	30 (6.0)
	2 to <4 hours	156 (31.5)
	4 to <6 hours	146 (29.4)
	6 to <8 hours	68 (13.7)
	≥8 hours	96 (19.4)
Sugar-sweetened beverage intake (<1 serving/day)		286 (57.7)
**Physical activity level**		
	Low	327 (65.9)
	Moderate	115 (23.2)
	High	54 (10.9)

**Table 2:** Spearman rank correlations among healthy behaviors of mothers with a preschool child (N=496).

Variable	1	2	3	4
1. Parent physical activity level	—	.335^a^	.012	–.026
2. Fruit and vegetable intake (≥5 servings/day)		—	.026	–.043
3. Sedentary screen time (<4 hours/day)			—	.150^a^
4. Sugar-sweetened beverage intake (<1 serving/day)				—

^a^*P*<.001.

**Table 3:** Independent *t* tests and chi-square tests of sociodemographic, intrapersonal, interpersonal, and home environment characteristics of participants by cluster (N=496).

Variable				Less healthy/inactive moms (n=280)		Health conscious/active moms (n=216)		*t*_494_		χ^2^_1_	*P*	
**Sociodemographic characteristics**												
	Age, mean (SD)			32.67 (5.57)		31.97 (5.81)		1.36			.17	
	Black or African American, non-Hispanic, n (%)			24 (8.6)		23 (10.7)				0.6	.43	
	White, non-Hispanic, n (%)			216 (77.1)		144 (66.7)				6.7	.01	
	Low education attainment (some college or less; % yes), n (%)			158 (56.4)		143 (66.2)				4.9	.03	
	**Maternal employment, n (%)**									6.5	.01	
		Do not work			167 (59.6)		104 (48.2)					
		Part- or full-time work			113 (40.4)		112 (51.9)					
	Number of children in household, mean (SD)			2.14 (0.91)		2.30 (1.13)		–1.77			.08	
	Family affluence score, mean (SD)			5.48 (1.58)		5.73 (1.55)		–1.73			.00	
	Food insecurity risk, mean (SD)			1.99 (1.96)		2.00 (1.80)		–0.09			.93	
**Health-related assessments**												
	Body mass index (BMI), mean (SD)			28.52 (8.46)		26.41 (6.71)		3.11			.002	
	Waist circumference, mean (SD)			35.81 (7.79)		33.38 (6.63)		3.74			<.001	
	Child BMI percentile (n=446),^a^ mean (SD)			61.27 (34.33)		66.71 (35.28)		–1.63			.10	
	General health status,^b^ mean (SD)			2.65 (0.83)		2.25 (0.86)		–5.21			<.001	
	Depression severity, mean (SD)			1.13 (1.48)		0.98 (1.45)		1.08			.28	
	Body dissatisfaction, mean (SD)			2.73 (1.11)		2.37 (1.09)		3.64			<.001	
	Primary relative with history of obesity (% yes), n (%)			118 (42.2)		63 (29.2)				8.9	.003	
**Intrapersonal characteristics**												
	**Maternal weight-related behaviors**											
		Physical activity level,^c^ mean (SD)			10.83 (8.19)		21.37 (8.64)		–13.87			<.001
		**Screen time,^c^mean (SD)**			306.8 (174.8)		311.53 (185.09)		–0.29			.77
			<4 hours/day, n (%)		105 (37.50)		81 (37.50)			0		>.99
		Sleep duration, mean (SD)			6.99 (1.36)		7.02 (1.62)		–0.24			.81
		Sleep quality, mean (SD)			3.13 (0.89)		3.40 (0.89)		–3.39			.001
	**Maternal dietary intake**											
		**Fruit and vegetable (servings/day),^c^ mean (SD)**			3.16 (1.23)		6.38 (1.78)		–22.73			<.001
			≥5 servings/day, n (%)		9 (3.2)		175 (81.0)			316.3		<.001
		Milk (servings/day), mean (SD)			3.06 (2.93)		4.96 (2.89)		–7.20			<.001
		**Sugar-sweetened beverage^c^ (servings/day), mean (SD)**			0.76 (0.71)		0.99 (0.98)		–2.89			.004
			<1 serving/day, n (%)		165 (58.9)		121 (56.0)			0.4		.52
	**Maternal eating styles, mean (SD)**											
		Disinhibited eating			1.94 (0.71)		1.97 (0.81)		–0.34			.74
		Emotional eating			2.14 (0.88)		1.97 (0.87)		2.12			.04
		Dietary restraint eating			2.36 (0.72)		2.53 (0.75)		–2.53			.01
	**Maternal self-perceptions, mean (SD)**											
		Personal organization (self-effectiveness)			3.55 (0.83)		3.82 (0.80)		–3.65			<.001
		Need for cognition			3.29 (0.98)		3.75 (0.92)		–5.34			<.001
		Parenting self-efficacy			3.99 (0.82)		4.23 (0.75)		–3.42			.001
		Stress management			3.87 (0.81)		4.02 (0.70)		–2.12			.03
		Stress management self-efficacy			2.49 (0.98)		2.81 (1.01)		–3.48			.001
	**Health behavior values, mean (SD)**											
		Importance of physical activity for self			3.17 (0.95)		3.92 (0.79)		–9.60			<.001
		Importance of physical activity for child			3.63 (0.86)		4.10 (0.79)		–6.30			<.001
		Encourages/facilitates child physical activity			4.10 (0.66)		4.40 (0.62)		–5.06			<.001
		Importance of modeling physical activity to child			3.93 (0.84)		4.41 (0.68)		–7.07			<.001
		Engages in physical activity with child frequently			3.06 (1.71)		4.40 (1.73)		–8.59			<.001
		Models physical activity to child frequently			2.69 (1.17)		3.59 (1.07)		–8.86			<.001
		Less frequency of modeling sedentary behaviors			2.81 (2.27)		2.87 (2.11)		–0.293			.770
**Social environment**												
	**Family meal patterns, mean (SD)**											
		Family meal frequency/week			13.04 (5.20)		14.67 (4.49)		–3.73			<.001
		Importance of family meals			4.48 (0.62)		4.61 (0.61)		–2.38			.02
		Positive family meal atmosphere			4.05 (0.84)		4.23 (0.86)		–2.33			.02
	**Family functioning and maternal engagement, mean (SD)**											
		Family support for healthy behaviors			4.47 (0.55)		4.31 (0.92)		2.22			.03
		Family conflict			1.88 (0.88)		1.81 (0.94)		0.87			.38
		Family cohesion			4.07 (0.75)		4.29 (0.66)		–3.52			<.001
**Home physical environment**												
	**Home environment: physical activity, mean (SD)**											
		Physical activity availability			3.63 (0.73)		3.96 (0.54)		–5.85			<.001
		Physical activity accessibility (n=524)			4.14 (1.21)		4.37 (0.99)		–2.28			.02
	**Media devices in the home, mean (SD)**			11.36 (4.02)		11.57 (4.23)		–0.56			.57	
		Daily screen time child allowed			557.09 (840.51)		380.14 (469.98)		2.97			.003
	**Home environment: food availability, mean (SD)**											
		Household fruit and vegetable availability (serving/person/day)			5.59 (2.28)		7.48 (2.23)		–9.25			<.001
		Household fatty/salty snack availability (serving/person/day)			7.41 (6.37)		9.52 (8.03)		–3.17			.002
		Household sugar-sweetened beverage availability (serving/person/day)			1.65 (1.55)		2.07 (2.02)		–2.55			.01

^a^Total children with biologically plausible data reported by mother (n=446; poor health/inactive moms: n=256, health conscious/active moms: n=190).

^b^Higher scores indicate poorer general health status.

^c^Variable included in cluster analysis.

Health conscious/active moms were significantly likely to be more physically active, have lower sedentary screen time, lower daily intake of sugar-sweetened beverages, and greater daily intake of fruits and vegetables compared to less healthy/inactive moms (Table 3). These two clusters were further validated when examining associations with maternal health status, weight, and intrapersonal, social environment, and the home physical environment characteristics.

There were no significant sociodemographic characteristic differences between the two clusters, except health conscious/active moms had a greater tendency to be employed (part-time/full-time) and be white, non-Hispanic. Less healthy/inactive moms were significantly more likely to have a higher BMI and waist circumference, and have a primary relative with a history of obesity compared to the other cluster. However, there were no significant differences between cluster groups on their child’s BMI percentile. Additionally, less healthy/inactive moms reported greater body dissatisfaction and poorer general health status compared to health conscious/active moms.

There were many intrapersonal characteristics that differed between the two clusters. For example, less healthy/inactive moms were significantly more likely to be have lower sleep quality and consume less daily servings of milk compared to health conscious/active moms. Additionally, health conscious/active moms were significantly more likely to have greater personal organization, need for cognition, parenting self-efficacy, and stress management self-efficacy compared to less healthy/inactive moms. Nearly, all health behavior value variables were significantly different between the two clusters. That is, health conscious/active moms were significantly more likely to place greater importance on physical activity for self and their child, and encourage and model physical activity for their child compared to less healthy/inactive moms.

The only social environment characteristics that differed between the two clusters was family meal frequency and family cohesion. That is, health conscious/active moms had significantly more family meals per week and more family cohesion compared to less healthy/inactive moms.

As anticipated, health conscious/active moms’ home physical activity environments had significantly greater availability of physical activity and stricter limits on children’s daily screen time compared to less healthy/inactive moms. Additionally, health conscious/active moms had greater availability in the home for fruits and vegetables, fatty/salty snacks, and sugar-sweetened beverages compared to less healthy/inactive moms.

## Discussion

### Principal Results

Overall, cluster analysis findings demonstrate two distinct patterns of obesity-related behaviors among mothers of preschool-aged children as evidenced by the external validation of clusters when examining associations with maternal health status, weight, intrapersonal, social environment, and the home physical environment characteristics. Comparing behavioral profiles of mothers of young children assigned to groups via cluster analysis adds qualitative insights, which may improve tailoring of interventions intended to effect behavior change. Thus, the two-cluster profiles found in our study may have implications for future obesity prevention interventions among families with young children.

Obesity-related behaviors were not strongly defined by sociodemographic characteristics, variables typically used to tailor nutrition interventions. Instead, a number of health-related variables and anthropometric markers (ie, weight status, waist circumference) were related to cluster membership. For instance, less healthy/inactive moms had a higher BMI, waist circumference, body dissatisfaction, and poorer general health status compared to health conscious/active moms. Given the negative health outcomes associated with membership in the less healthy/inactive moms cluster and mothers’ influence on their children living in the same home environments [70], interventions that focus on improving obesity-related risk behaviors of mothers with young children are warranted.

The four obesity-related variables (ie, mother’s physical activity level, sedentary screen time, daily fruit and vegetable intake, and sugar-sweetened beverage intake) used to define clusters in this study may be useful markers for tailoring obesity interventions. However, regardless of cluster groupings, a large proportion of participants were still not meeting the recommended daily intakes of fruits and vegetables along with suggested limits of daily sedentary screen time and sugar-sweetened beverage intake [67]. Further examination of cluster groupings by intrapersonal, social environment, and home physical environment characteristics illustrates the complexity of obesity and potential target areas for influencing behavior change among mothers with preschool-aged children. This study found cluster grouping associations at all levels; however, more significant associations were found at the intrapersonal and home physical environment levels suggesting parents might be strong influencers of their child’s behaviors.

### Comparison With Prior Work

Prior research has found concordance of clustering patterns between children and their mothers suggesting that modeling of obesity-related risk behaviors by parents may be particularly important influences on children’s behavior [70]. Although not formerly tested in this study, health conscious/active moms reported significantly greater health behavior values on the importance of modeling physical activity for their child and placing limits on child sedentary screen time compared to less healthy/inactive moms. Both physical activity levels and eating behaviors of parents are predictive of obesity in children [72]. Additionally, prior research has found a positive relationship between family support and increased physical activity along with family-based obesity treatment programs being the most effective at combating pediatric obesity [73]. Thus, obesity interventions that are family based may be more effective when encouraging parents to improve their own obesity-related risk behaviors along with developing the parenting skills needed to model these same healthy behaviors for their children.

Over time, diets have changed dramatically during the preschool period with an increase intake of added sugars that persists into adolescence [74]. Interestingly, health conscious/active moms had greater home availability of fruits and vegetables per family member, but also greater home availability of low nutrient-dense foods, such as sugar-sweetened beverages and salty/fatty snacks. Having greater availability of these food items does not necessarily correlate with greater food consumption of these low nutrient-dense food items. Future research should further explore whether greater availability of high nutrient-dense versus low nutrient-dense foods in the home has any direct effect on food intake and, in turn, obesity risk.

### Limitations

Taking a simplistic approach can obscure true interrelationships between health behaviors [75]. Considering the complexity of behaviors is important when developing obesity prevention interventions; however, it is also important to note that cluster analysis is an exploratory technique. The cluster profiles observed in our study may have differed with the inclusion of different variables, clustering algorithm, or sample. Other limitations are the cross-sectional design that limits the ability to make interferences of causality from the observed associations, and the potential for reporting error and bias given all information was self-reported by participants. Although the sample included mothers of preschool-aged children who had demographics similar to the overall US population, findings may not be generalizable to fathers or families with children of different ages and in other countries.

### Conclusions

Despite these study limitations, this is one of only a few studies that have examined the clustering of obesity-related risk behaviors in mothers of preschool-aged children. Additionally, this study took a socioecological approach to comprehensively examine an array of obesity-related risk behavior factors among mothers with preschool-aged children using reliable and validated measures. Given the strong influence mothers have on their child’s weight-related behaviors during the preschool years and subsequent years following into adulthood [15-19], study findings further suggest targeting obesity prevention interventions using a socioecological approach that encourages mothers to model positive health behaviors for their children at the intrapersonal, social environment, and home physical environment levels. Future research assessing the value of this socioecological approach is warranted.

## Appendix

Multimedia Appendix 1 Descriptive Statistics of Sociodemographic, Intrapersonal, Interpersonal and Home Environment Characteristics of Participants (N=496).

## Abbreviations

BMI: body mass index
HOMES: Home Obesogenic Measure of Environments
