# Supplementary material for: Clustering of Obesity-Related Risk Behaviors Among Families With Preschool Children Using a Socioecological Approach: Cross-Sectional Study
Source: JMIR Pediatr Parent. 2018 Apr 25;1(1):e10320. doi: 10.2196/10320 (PMC6716486; doi:10.2196/10320)
Supplement: Multimedia Appendix 1 [file pediatrics_v1i1e10320_app1.pdf]

Descriptive statistics of sociodemographic, intrapersonal, interpersonal, and home environment characteristics of participants (N=496).

Variables	# Items (possible score range)	Scale type	Cronbach alpha[a]	Mean (SD) or n (%)
**Sociodemographic characteristics**				
Age	1	Years		32.36 (SD 5.68)
Black or African American, non-Hispanic	1	Yes/no		47 (9.5%)
White, non-Hispanic	1	Yes/no		360 (72.6%)
Low education attainment (some college or less) (% yes)	1	Yes/no		301 (60.7%)
**Maternal employment**	1	Categorical response		
Do not work				271 (54.6%)
Part- or full-time work				225 (45.4%)
Number of children in household	1	Total #		2.21 (SD 1.01)
Family affluence score	3 (0-9)	Varies per item		5.59 (SD 1.57)
Food insecurity risk	2 (1-4)	4-Point agreement rating	.84	2.00 (SD 1.89)
**Health-related assessments**				
Body mass index (BMI)	1	Self-report height and weight		27.60 (SD 7.81)
Waist circumference	1	Self-report measurement (inches)		34.75 (SD 7.40)
Child BMI percentile (n=446)[b]	1	Self-reported height and weight by mother		63.58 (SD 34.80)
General health status[c]	1	5-Point excellence rating[d]		2.94 (SD 4.58)
Depression severity	2 (1-4)	4-point frequency rating[e]	.81	1.06 (SD 1.46)
Body dissatisfaction	1 (1-4)	4-point frequency rating[e]		2.57 (SD 1.11)
Primary relative with history of obesity (% yes)	1	Yes/no		181 (36.5%)
**Intrapersonal characteristics**				
**Maternal weight-related behaviors**				
Physical activity level[f]	3 (0-7)	8-Point exercise frequency scale[g]		15.42 (SD 9.88)
**Screen time[f]**	1	Minutes/day		308.86 (SD 179.18)
<4 hours/day	1	Yes/no		186 (37.5%)
Sleep duration	1	Hours/day		7.00 (SD 1.48)
Sleep quality	1 (1-5)	5-Point excellence rating[h]		3.25 (SD 0.90)
**Maternal dietary intake**				
**Fruit and vegetable (servings/day)[f]**	10 (0->1)	6-Point servings eaten[i] scale		4.56 (SD 2.19)
≥5 servings/day	1	Yes/no		184 (37.1%)
Milk (servings/day)	1 (0->1)	9-Point servings drank[j] scale		3.89 (SD 3.06)
**Sugar-sweetened beverage[f] (servings/day)**	4 (0->1)	9-Point servings drank[j] scale		0.86 (SD 0.85)
<1 serving/day	1	Yes/no		286 (57.7%)
**Maternal eating styles**				
Disinhibited eating	3 (1-4)	4-Point agreement rating[k]	.81	1.95 (SD 0.75)

Emotional eating	3 (1-4)	4-Point agreement rating[k]	.75	2.06 (SD 0.88)
Dietary restraint eating	4 (1-4)	4-Point agreement rating[k]	.74	2.43 (SD 0.74)
**Maternal self-perceptions**				
Personal organization (self-effectiveness)	4 (1-5)	5-Point agreement rating[l]	.69	3.67 (SD 0.83)
Need for cognition	1 (1-5)	5-Point agreement rating[l]		3.49 (SD 0.98)
Parenting self-efficacy	1 (1-5)	5-Point agreement rating[l]		4.09 (SD 0.80)
Stress management	2 (1-5)	5-Point agreement rating[l]	.84	3.93 (SD 0.77)
Stress management self-efficacy	1 (1-5)	5-Point agreement rating[l]		2.63 (SD 1.00)
**Health behavior values**				
Importance of physical activity for self	3 (1-5)	5-Point agreement rating[l]	.82	3.49 (SD 0.96)
Importance of physical activity for child	3 (1-5)	5-Point agreement rating[l]	.68	3.83 (SD 0.86)
Encourages/facilitates child physical activity	5 (1-5)	5-Point agreement rating[l]	.88	4.23 (SD 0.66)
Importance of modeling physical activity to child	2 (1-5)	5-Point agreement rating[l]	.79	4.14 (SD 0.81)
Engages in physical activity with child frequently	2 (0-7)	8-Point frequency scale[m]		3.64 (SD 1.84)
Models physical activity to child frequently	2 (0-7)	8-Point frequency scale[m]		3.09 (SD 1.21)
Less frequency of modeling sedentary behaviors	2 (0-7)	8-Point frequency scale[m]		2.84 (SD 2.20)
**Social environment**				
Family meal patterns				
Family meal frequency/week	3 (0-21)	0-7 Days for breakfast, lunch, dinner; score is sum of 3 meals		13.75 (SD 4.96)
Importance of family meals	3 (1-5)	5-Point agreement rating[l]	.70	4.53 (SD 0.62)
Positive family meal atmosphere	3 (1-5)	5-Point agreement rating[l]	.70	4.13 (SD 0.85)
**Family functioning and maternal engagement**				
Family support for healthy behaviors	4 (1-5)	5-Point agreement rating[l]	.81	4.40 (SD 0.74)
Family conflict	2 (1-5)	5-Point agreement rating[l]	.82	1.85 (SD 0.90)
Family cohesion	3 (1-5)	5-Point agreement rating[l]	.84	4.16 (SD 0.72)
**Home physical environment**				
**Home environment: physical activity**				
Physical activity availability	12 (1-5)	5-Point agreement rating[l]	.72	3.77 (SD 0.68)
Physical activity accessibility (n=524)	2 (1-5)	5-Point agreement rating[l]	.90	4.24 (SD 1.12)

**Media devices in the home**	6	Total devices		11.46 (SD 4.11)
Daily screen time child allowed	1	Minutes/day		480.03 (SD 708.40)
**Home environment: food availability**				
Household fruit and vegetable availability (serving/person/day)	10 (0->1)	9-Point servings scale[J]		6.41 (SD 2.44)
Household fatty/salty snack availability (serving/person/day)	4 (0-32)	9-Point servings scale[J]		8.33 (SD 7.21)
Household sugar-sweetened beverage availability (serving/person/day)	4 (0->1)	9-Point servings scale[J]		1.83 (SD 1.78)
Household sugar-sweetened beverage availability (serving/person/day)	4 (0->1)	9-Point servings scale[J]		1.83 (SD 1.78)

[a]For variables without value, Cronbach alpha was not appropriate given the scale type or <2 items.

[b]Total children with biologically plausible data reported by mother (n=446)

[c]Higher scores indicate poorer general health status.

[d]5-point excellence rating: poor, fair, good, very good, excellent.

[e]4-point frequency rating: not at all, several days, more than half the days, nearly every day.

[f]Variable included in cluster analysis.

[g]8-point exercise frequency: days/week for items scored 0, 1, 2, 3, 4, 5, 6, and 7; days/week weighted by exercise intensity (weights of 1, 2, 3 for walking, moderate, and vigorous activity, respectively) and summed to create scale score with higher scores indicating greater activity level.

[h]5-point excellence rating: very bad, bad, okay, good, very good.

[i]Scale scoring Is protected by copyright.

[j]9-point serving: <1 time/week, 1 day/week, 2 days/week, 3 days/week, 4 days/week, 4 days/week, 6 days/week, 7 days/week, >1 time/day.

[k]4-point agreement scale: definitely false, mostly false, mostly true, definitely true.

[l]5-point agreement scale: strongly disagree, disagree, neither agree or disagree, agree, strongly agree.

[m]8-point frequency scale: 0 (almost never), 1, 2, 3, 4, 5, 6, and 7 days per week.
